# UrbangEnCy: An emergency events dataset based on citizen sensors for monitoring urban scenarios in Ecuador

**DOI:** 10.1016/j.dib.2020.106693

**Published:** 2020-12-24

**Authors:** Jorge Parraga-Alava, Roberth Alcivar-Cevallos, Leticia Vaca-Cardenas, Jaime Meza

**Affiliations:** a Facultad de Ciencias Informáticas, Universidad Técnica de Manabí, Avenida Jose María Urbina, Portoviejo 130104, Ecuador; b Departamento de Ingeniería Informática, Universidad de Santiago de Chile, Av. Ecuador 3659, Santiago 9160000, Chile

**Keywords:** Ecuador, Emergency events, Citizen sensors, ECU 911, Social media, Text mining

## Abstract

Recently, the use of the citizen-sensors (people generating and sharing real data by social media) for detecting and disseminating emergency events in real-time have shown a considerable increase because people at the place of the event, as well as elsewhere, can quickly post relevant information on this type of alerts. Here, we present an emergency events dataset called *UrbangEnCy*. The dataset contains over 25500 texts in Spanish posted on Twitter from January 19th to August 19th, 2020, with emergencies and non-emergencies related content in Ecuador. We obtained, cleaned and, filtered these tweets and, then we selected the location and temporal data as well as tweet content. Besides, the data set includes annotations regarding the type of tweet (emergency / non-emergency) as well as additional nomenclature used to describe emergencies in the Center for immediate response service to emergencies (ECU 911) of Ecuador and international emergency services agencies (ESAs). *UrbangEnCy* dataset facilitates evaluating data science performance, machine learning, and natural language processing algorithms used with supervised and unsupervised problems re- related to text mining and pattern recognition. The dataset is freely and publicly available at https://doi.org/10.17632/4x37zz82k8.

**Specifications Table**

**Table:** 

Subject	Data Science
Specific subject area	Text and social media mining of emergencies-related events for designing, implementing and, evaluating predictive and descriptive models.
Type of data	Dataset in delimiter-separated values format (DSV).
How data were acquired	The Twitter API search along with *rtweet* R package.
Data format	Raw Processed and labelled DSV format.
Parameters for data collection	Tweets were collected using Twitters Streaming API, considering two types of filters in the query. The location filtering thought tweets within a bounding circumference of 600 miles of radius according to geographical coordinates -1.83,-78.18 covering continental Ecuador. The keyword filtering included at least one of the most frequently used words to refers to emergency events in Ecuador, including ǣdesmayoǥ, ǣaccidenteǥ,ǣasaltoǥ, ǣatropelloǥ, ǣemergenciaǥ, ǣmuerteǥ, victimaǥ, ǣconsumo drogaǥ, ǣheridoǥ, ǣmano armadaǥ and others. The location and keyword filtering looks for tweets containing such queries and posted by users with public profiles located anywhere in Ecuador.
Description of data collection	Tweets on non-emergency and emergency events were retrieved using a set of Spanish keywords frequently employed to refer to urban emergencies. The Twitter API and the *rtweet* R package were used to collect posts on Twitter from January 19th to August 19th, 2020. Annotation data were manually generated by five human annotators considering four types of emergency categories/levels frequently used in Ecuador’s integrated security service (ECU 911) and international Emergency Services Agencies(ESAs).
Data source location	City/Town/Region: All regions except Galapagos Islands Country: Ecuador Latitude and longitude: -1.83,-78.18 with a radius of 600 miles.
Data accessibility	Repository name: urbangEnCy Data identification number: DOI: 10.17632/4x37zz82k8 Direct URL to data: https://data.mendeley.com/datasets/4x37zz82k8 We do not provide the tweet text, posted date, or geographical location to accomplish with Twitter terms and conditions, but we share the IDs so that the tweets can be downloaded from the Twitter API.

## Value of the Data

• This dataset can help analyze the integrity of emergency alerts reported by social media users and train and evaluate classification and event detection techniques for real-time disaster and crisis management applications.
• Researchers in data science, machine learning, and natural language processing fields can utilize these data to train supervised and unsupervised models to analyze and detect sentiments such as sarcasm, irony, or satire in Spanish texts that use words commonly associated with emergencies.
• Data can be used in Natural Language Processing (NLP) area for fake events detection on Twitter since a large number of posts about events that contain commonly used Spanish words refer to emergencies but actually, they are not.
• The dataset includes annotations of the four categories of emergencies according to Ecuadorian and International terminology, which can be used to improve the performance of classification/clustering algorithms trained on this dataset as well as to extract new knowledge about geographical behavior of the urban emergencies event in Ecuador.
• Data can serve as a motivation to encourage further research into social media analysis in Spanish texts and thereby to improve the accuracy and precision levels of machine learning and data science algorithms applied to data related to this language.

## 1 Data Description

The dataset provides tweets posted by citizen sensors on Twitter. These posts contain information about possible emergency events reported in Ecuador during January and August 2020. In total, nine variables and 25547 instances (tweets) are included in the data set and detailed in Table 1.

**Table 1:** Description of variables available in UrbangEnCy dataset.

Variable	Description	Type	T/A
ID	It is a unique identifier for each tweet.	Identifier	A
crated_at	It is the date and hour when the tweet was posted.	Character^a^	T
text	It is the content of the tweet.	Character	T
place_name	It is the city reported in the users profile who posted the tweet.	Character	T
center_name	It corresponds to the ECU 911 Center, where the place is located.	Categorical	A
category1	It indicates if the tweet really corresponds to an alert of emergency or not.	Categorical	A
category2	It indicates the articulated institution that should address the emergency reported in the tweet.	Categorical	A
category3	It indicates the most common specific emergencies in Ecuador, according to each articulated institution.	Categorical	A
category4	It indicates the appropriate response agency that will mobile the available resources to provide immediate attention to citizens. This category is the most commonly used in emergency services centers worldwide.	Categorical	A

a In format: yyyy-mm-dd hh:mm:ss. Note that variables highlighted in gray are not available in the final version of the dataset due to Twitter data policies (See Ethics Statement section).

Table 1 shows the descriptions of the variables as well as the type of values for each one. The last column indicates whether the data value was obtained from Twitter (T) o added (A) as part of the dataset creation. Note that the social network information includes variables related to the tweet, its posting date, and the city where it originated. At this point, it is also worth noticing that the variable “*center_name*” has 14 levels representing each ECU 911 Centers. Finally, the levels for variables “*category1*”, “*category2*”, “*category3*”, and “*category4*” are shown in Tables 2-3.

**Table 2:** Levels of the emergency classes-related variables according to international ESAs nomenclature.

Main level^a^	Low level^b^	Count	%
emergencia	bomberos	853	57.2%
	EMS	92	6.2%
	policía	546	36.6%
no emergencia	no emergencia	24056	100%

a category1,

b category4.

**Table 3:** Levels of the emergency classes-related variables according to ECU 911 nomenclature.

Main level^a^	Intermediate level^b^	Low level^c^	Count	%
	gestión de riesgos	amenazas naturales	32	2.1%
	gestión de siniestros	accidente de tránsito	323	21.7%
		asistencia	66	4.4%
		eventos clínicos	2	0.1%
		incendios	271	18.2%
		transporte secundario	4	0.3%
		accidente de tránsito	7	0.5%
	gestión sanitaria	eventos clínicos	19	1.3%
		transporte secundario	60	4.0%
		accidente de tránsito	25	1.7%
		actos inmorales	143	9.6%
emergencia	seguridad ciudadana	presencia policial	112	7.5%
		robo	176	11.8%
		servicios	62	4.2%
	servicio militar	control de armas y explosivos	3	0.2%
		alerta/seguridad	23	1.5%
	servicios municipales	energía elȨctrica	50	3.4%
		mantenimiento en vías pȦblicas	28	1.9%
		servicios	1	0.1%
	tránsito y movilidad	accidente de tránsito	74	5.0%
		servicios	10	0.7%
no emergencia	no emergencia	no emergencia	24056	100%

a category1,

b category2,

c category3.

For each *place_name*, the associated ECU 911 center list has been drawn up in *center_name* according to its geographical proximity. For a given tweet, it is included whether or not it is a real emergency event in *category1*. If a tweet is a real emergency event, it is classified by both ESAs and ECU 911 nomenclatures into *category4, category2*, and *category3* variables, respectively. If a tweet is an unreal emergency event, the values for such categories are denoted as *”no emergencia”*.

In Tables 2-3 the frequency of real and unreal emergencies is reported for each category considered in the dataset. Note that the real emergencies correspond only to 1491 tweets, and for each emergency, there are levels of detail according to the International (ESA) and Ecuador (ECU 911) nomenclature.

In Table 2, the main and low levels correspond to variables “*category1*” and “*category4*”, according to ESAs nomenclature. Here, the emergencies that require the presence of firefighters (*bomberos*) (57.2%) stand out, followed by those that need police personnel (*policía*) (6.2%) and finally those related to EMS (6.2%). While the tweets that are unreal emergencies, they are equivalent to 100% since they do not have sub levels.

In Table 3, the main, intermediate, and low levels correspond to variables “*category1*”, “*category2*”, and “*category3*”, according to ECU 911 nomenclature. *Category2* reports the articulated institution that should address the emergency declared in the tweet. They are obtained from https://www.ecu911.gob.ec/instituciones-articuladas/. In Table 3, the highest percentage (21.7%, 18.2%, and 11.8%) of tweets that are truly emergencies correspond to *accidentes de tránsito, incendios* and *robo*, respectively. In contrast, for tweets with an unreal emergency for the international nomenclature case, 100% of them have the value *no emergencia*.

**Fig. 1:**
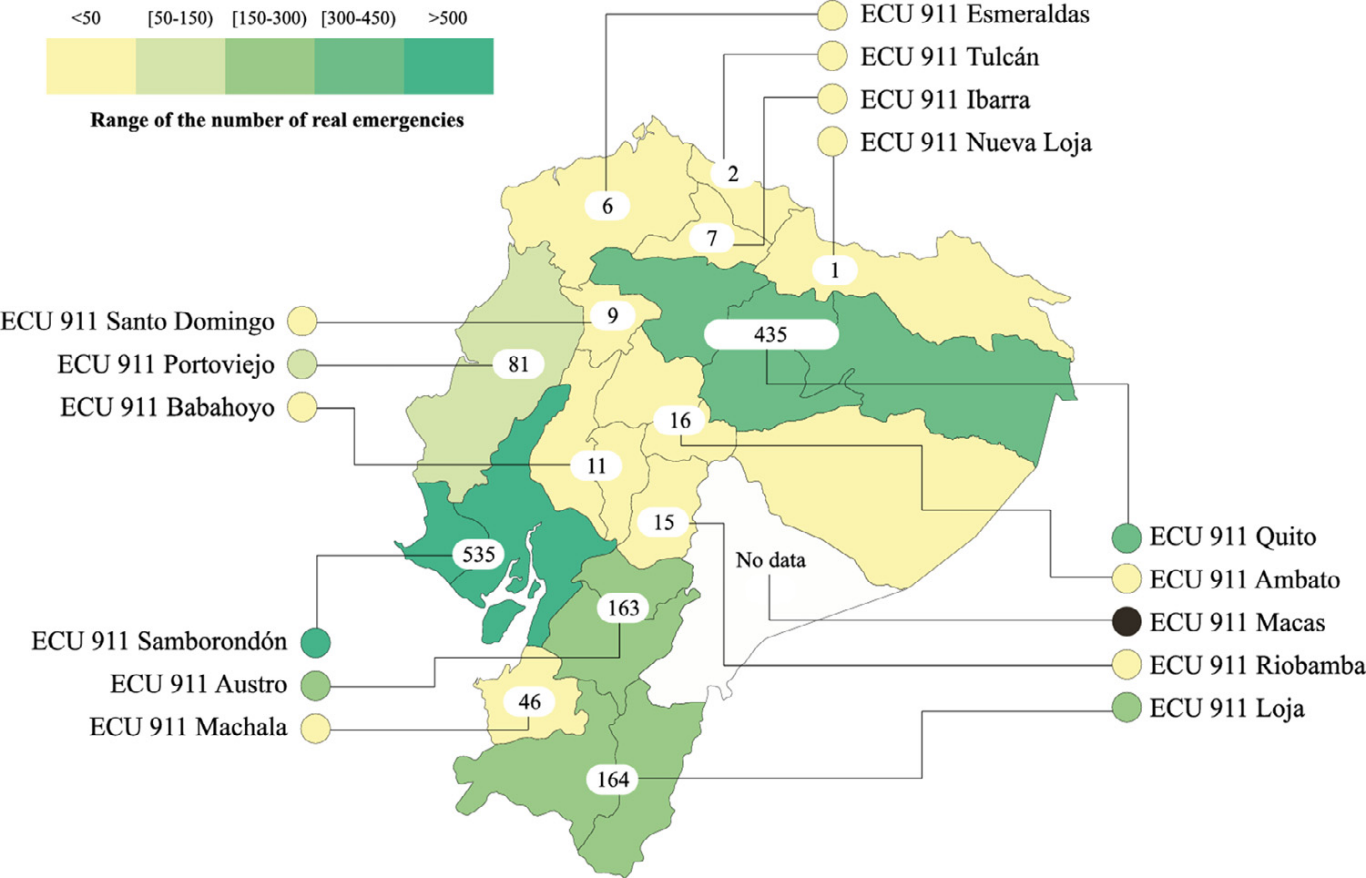
Number of real events emergency reported in the dataset for each geographical location of the ECU 911 Centers. (For interpretation of the references to color in this figure, the reader is referred to the web version of this article.)

Furthermore, Fig. 1 shows the ECU Centers distribution that should deal with the emergency for each one of these emergencies.

Fig. 1 shows the geographical distribution of the reported emergency events in the gathered tweets. We noticed that most emergency events were reported by citizen sensors with Twitter accounts located in the ECU 911 Centers of Samborondón and Quito. Fig. 1 also shows that the number of declared emergencies are low (light yellow color) in most ECU 911 centers. In contrast, in only two centers (Austro and Loja), it exceeded 100 emergencies during the study period.

## 2 Experimental Design, Materials and Methods

The tweets about possible emergency events were acquired using Twitter’s Streaming API, consuming the information posted on Twitter by citizen sensors. The dataset construction process consisted of two stages: data acquisition and annotation.

### 2.1 Data acquisition

**Fig. 2:**
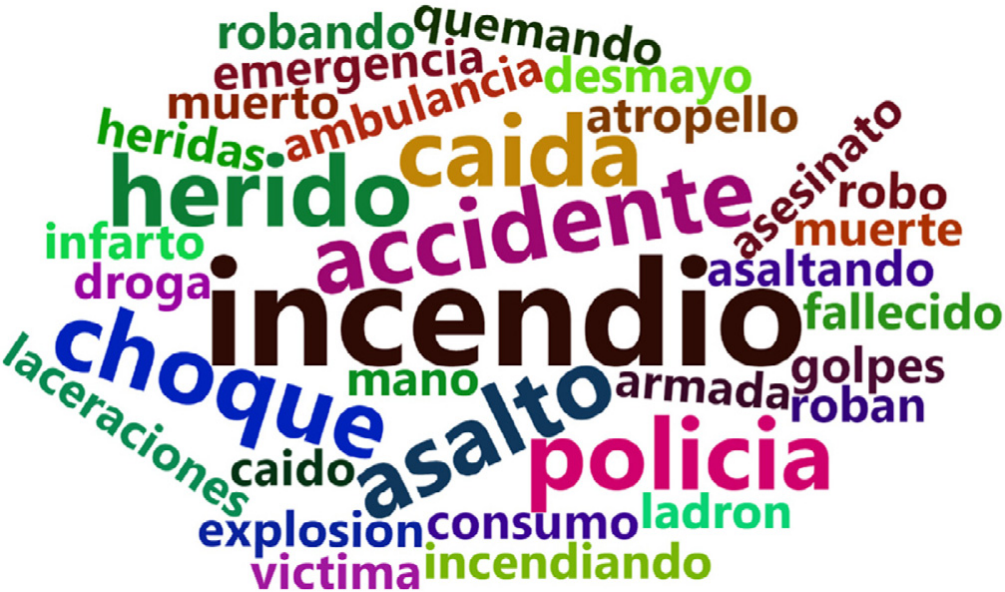
Wordcloud of the query (in Spanish) used to acquire the possible emergency events reported by citizen sensors tweets.

In this stage, tweets on no-emergency and emergency events were retrieved using the *rtweet* R package. For this purpose, the Twitter API search was run from January 19th to August 19th, 2020, considering a set of Spanish keywords shown in Fig. 2. The set of Spanish keywords was created considering the words used in events reported through emergency calls in the ECU 911 centers during 2018-2019. Note that due to Twitter API limitations, the set number of elements should be a character string that does not exceed a maximum of 500 characters. A term-document matrix was used to achieve this, and then the words that occur most frequently and that adds up to 500 characters were selected.

Fig. 2 shows the frequently used words to refer to urban emergencies in Ecuador. They are used to get tweets data on statuses identified via the search query that also included the geographical coordinates -1.83,-78.18 with a radius of 600 miles to ensure that the tweets obtained correspond to those generated in Ecuador. Note that this query was configured to receive only tweets posted by users with a public profile and not other types of posts such as retweets or likes.

Over the query’s original data, we carried out a pre-processing procedure where emoticons within the tweets and about 50 tweets generated in the Galapagos Islands were eliminated, which finally yielded a total of three variables: “*created_at,*” “*text,*” and “*place_name*”. The“*ID*” variable was added after that, assigning an identifier from 1 to the total number of tweets, 25547. Finally, the variable “*place_name*” was incorporated, making a match between the “*place_name*” where the tweet was generated, and the ECU 911 Centers’ coverage area reported in the official web site^1^ of the institution.

### 2.2 Annotation process

In this stage and to enrich the data set and make it useful for research in machine learning, data mining, or related areas, four emergency categories were incorporated for each tweet. As in Ecuador, the ECU 911 is the leading Center for immediate and comprehensive response service to emergencies in such territory. Three categories related to the emergency events reported by this Center were added, and one category according to international ESAs nomenclature. To assign each category’s values, five annotators manually inspected the tweets and did it. The values for each one are shown in Table 4.

**Table 4:** Interpretation of Cohen’s kappa.

Kappa	Level of agreement	% of data reliability
0-0.20	None	0-4%
0.21-0.39	Minimal	4-15%
0.40-0.59	Weak	15-35%
0.60-0.79	Moderate	35-63%
0.80-0.90	Strong	64-81%
Above 0.90	Almost Perfect	82-100%

To validate the annotations’ consistency, the agreement between the annotations carried out for the same category but by different annotators was calculated. The idea of this is to observe if the annotators match in the category assigned to each tweet. Inspired by works [1] and [2], the interpretation of Kappa coefficient suggested by McHugh [3] was used to measure the agreement. It can be simplified in Table 4 as follows:

In Table 4, any kappa value below 0.60 indicates inadequate agreement among the annotators and, little confidence should be placed in the labeling process. Here, % of data reliability corresponds to the squared Kappa value, an equivalent of the squared correlation coefficient. The level of agreement obtained by our annotators was almost perfect since the Kappa value was of 96%, 95%, 95% and, 96% for “*category1*”, “*category2*”, “*category3*”, and “*category4*”, respectively.

The final value of each category (label) was selected using a *plurality strategy*. Note that it differs from majority strategy because, for instance, if the annotators assign to a tweet the values of a category as A, B, C, C, D, then C is selected as plurality value but not the majority value (because it occurs only 2/5 of the times, and majority implies > 1/2 of the times). In cases of times, the value is arbitrarily selected in random order.

The R/R Studio software was used to perform data acquisition and pre-processing procedures. The software was run using a standard computer (Intel (R) Core (TM) i7-6500U, CPU @2.50 GHz, 8 GB RAM).

## Ethics Statement

According to Twitters data policies, *UrbangEnCy* does not provide any personally identifiable information, and only the tweet IDs and human-annotated variables and labels are shared. Further information regarding Twitters Developer Agreement and Policy is available in the official documentation accessible at https://developer.twitter.com/en/developer-terms/agreement-and-policy.

## CRediT Author Statement

**Jorge Parraga-Alava:** Conceptualization, Methodology, Software, Validation, Formal analysis, Investigation, Resources, Data curation, Writing - original draft, Writing - review & editing, Supervision, Funding acquisition, Project administration. **Roberth Alcivar-Cevallos:** Data curation, Investigation, Writing - original draft, Writing - review & editing. **Leticia Vaca-Cardenas:** Investigation, Writing - original draft, Writing - review & editing. **Jaime Meza:** Writing - original draft, Writing - review & editing, Funding acquisition.

## Declaration of Competing Interest

The authors declare that they have no known competing financial interests or personal relationships which have or could be perceived to have influenced the work reported in this article.

## Footnotes

1 https://www.ecu911.gob.ec/cobertura-nacional/
